# Reducing SO_2_ Doses in Red Wines by Using Grape Stem Extracts as Antioxidants

**DOI:** 10.3390/biom10101369

**Published:** 2020-09-25

**Authors:** Irene Esparza, Blanca Martínez-Inda, María José Cimminelli, Maria Carmen Jimeno-Mendoza, José Antonio Moler, Nerea Jiménez-Moreno, Carmen Ancín-Azpilicueta

**Affiliations:** 1 Department of Sciences, Universidad Pública de Navarra, Campus Arrosadía s/n, 31006 Pamplona, Spain; irene.esparza@unavarra.es (I.E.); blancamartinezinda96@gmail.com (B.M.-I.); cimminelli.120171@e.unavarra.es (M.J.C.); 2 Institute for Advanced Materials (INAMAT2), Universidad Pública de Navarra, 31006 Pamplona, Spain; 3 Navarra Viticulture and Oenological Research Station (EVENA), C/Valle de Orba, 34, 31390 Olite, Navarra, Spain; mc.jimeno.mendoza@unavarra.es; 4 Department of Statistics and Operational Research, Universidad Pública de Navarra, Campus Arrosadía s/n, 31006 Pamplona, Spain; jmoler@unavarra.es

**Keywords:** SO_2_ reduction, antioxidant activity, grape stem extract, polyphenolic compounds, sensory analysis

## Abstract

SO_2_ is a very important wine preservative. However, there are several drawbacks associated with the use of SO_2_ in wine. The aim of this work is to evaluate the effect of the partial substitution of SO_2_ in the Tempranillo wine by a Mazuelo grape stem extract and by a commercial vine wood extract (Vinetan^®^). The results were compared with a control sample (with no addition of any extract). After 12 months of storage in a bottle, total anthocyanin content, together with total polyphenol and flavonoid content were slightly higher for control wines than for those treated with extracts. These differences were of little relevance, as no differences in antioxidant activity were found between any of the wines at the end of the study. The sensory analysis revealed that the use of both extracts as partial substitutes of SO_2_ could lead to wines with good organoleptic properties, similar or even better to the control ones.

## 1. Introduction

Sulphur dioxide is used as an antioxidant and antimicrobial agent in food processing and is the most widely used preservative in the wine industry. In addition to direct oxygen removal, SO_2_ can act as an antioxidant by reacting with hydrogen peroxide and by reducing quinones to their phenol form [1,2]. On the other hand, sulphur dioxide acts against enzymatic and non-enzymatic oxidation of wines [3,4]. In red wine, the addition of SO_2_ intervenes favourably in the dissolution of mineral substances, the extraction of organic acids, and especially in the extraction of phenolic compounds (anthocyanins and tannins) responsible for the colour of red wines [3]. However, a very important role of this compound lies in its antimicrobial action against acetic and lactic acid bacteria as well as against moulds, preventing the wine deterioration and favouring its microbiological stabilization [5]. Furthermore, the addition of SO_2_ to the wine before the alcoholic fermentation exerts a selective antimicrobial activity against undesirable yeasts by inhibiting their growth and favouring the rapid development of *Saccharomyces cerevisiae* [6].

However, there are several drawbacks associated with the use of SO_2_ in wine such as the toxicity and the unpleasant odour in case of excess [7,8]. Excess SO_2_ deteriorates the organoleptic quality of the wine because it can produce undesirable flavours and a burning sensation after tasting [3]. In addition, several sulphur volatile compounds are related to undesirable reductive odours in wine [9,10,11] and three of these compounds were observed at concentrations above their threshold in faulted wines: hydrogen sulphide (H_2_S), methanethiol (MeSH), and dimethyl sulphide (DMS) [12]. On the other hand, many people are sensitive to sulphites and can develop different symptoms such as bronchospasm, bradycardia, dermatitis, urticaria, abdominal pain, diarrhoea, hypotension, shock, allergic responses with increasing risk of asthmatic attacks, trouble breathing, skin rashes, and stomach pain [13,14]. It is estimated that around 1% of the population has some clinical sensitivity to this food preservative, with increasing risk in asthmatic people [14,15].

Consequently, although the addition of SO_2_ is currently an essential treatment in winemaking, one of the main challenges for the wine industry is to find alternatives to this preservative for its replacement or reduction. Different physical and chemical methods alternative to SO_2_ have been studied [7]. Among the physical methods, the use of pulsed electric fields [4,16], high hydrostatic pressure [17], ultraviolet radiation [18], and ultrasounds [19] have been evaluated. The main drawback of these physical techniques is their lack of antioxidant activity, although they have a strong antimicrobial action. Regarding the addition of alternative chemicals, different doses of lysozyme and dimethyl dicarbonate [20], colloidal silver [21], and hydroxytyrosol [22] have been tested.

In the last years, special attention has been paid to the use of phenolic compounds as an alternative to sulphites [23,24]. In this sense, the exploitation of by-products from the winemaking industry to obtain extracts that can be used as natural preservatives seems very interesting. Winemaking by-products such as grape pomace, seeds, and grape stems are very rich in phenolic compounds, and these biomolecules with proper management could constitute valuable sources of natural preservatives as alternatives to sulphur dioxide [25,26]. Among these by-products, grape stems, which account for 3–7% of the bunch weight [3], are the least used at present. This residue is removed prior to the winemaking process to avoid a negative effect on wine organoleptic characteristics. The presence of grape stems during fermentation increases the wine astringency, mainly due to its high content of proanthocyanidins [27]. Grape stems have a high content in phenolic compounds, biomolecules with strong antioxidant activity that can effectively act as radical scavengers [28]. Phenolic acids, flavan-3-ols, flavonoids, and stilbenes are the main polyphenols in grape stems [27]. Moreover, these bioactive compounds also have an important antimicrobial action [29,30]. Antimicrobial activities of phenolic compounds have different mechanisms of action that have been recently reviewed [31,32]. Among all the mechanisms reported, the most important are the interaction with the cell membrane, which increases its permeability and causes its disruption; the inhibition of biofilm formation; the inhibition of the synthesis of peptidoglycan, which is an essential component of the bacterial cell wall; the inhibition of nucleic acid synthesis; the inhibition of electron transport chain and ATP synthesis; and the inhibition of bacterial enzymes and substrate deprivation. Likewise, García-Ruiz et al. [23] studied the effect of different families of polyphenols on several wine lactic acid bacteria: *Oenococcus oeni, Lactobacillus hilgardii*, and *Pediococcus pentosaceus*. They found that flavonoids and stilbenes showed the greatest inhibitory effects on the growth of the lactic acid bacteria strains analysed. On the other hand, hydroxycinnamic acids and hydroxybenzoic acids and esters had a medium inhibitory effect, and phenolic alcohols and flavan-3-ols showed the least effect.

Considering the above, the use of grape stems extracts as an alternative to sulphur dioxide, in addition to responding to the growing consumer demand for food free from chemical additives, allows us to face another of the current challenges of the agri-food industry, the waste management through the valorisation of by-products. Very few studies analyse the effect of substituting SO_2_ for extracts obtained from winemaking by-products. Raposo et al. [33] used a commercial grapevine-shoot extract (Vineatrol^®^) as a preservative in Syrah wines and Ruiz-Moreno et al. [34] proposed the utilization of a grape stem extract as an alternative to sulphur dioxide after analysing its in vitro antimicrobial and antioxidant effect. Therefore, the aims of this work are: (a) to evaluate the effect of the partial substitution of SO_2_ by Mazuelo grape stem extracts on the antioxidant capacity and phenolic profile during the winemaking process; (b) to compare the results obtained with those of a commercial vine wood extract (Vinetan^®^) and a control wine (with no addition of any extract). In addition, a simple sensory analysis was performed to check the organoleptic quality of all the wines thus obtained.

## 2. Materials and Methods

### 2.1. Extracts

The grape stem extract was obtained through an extraction using GRAS solvents and stems of grapes from the Mazuelo variety harvested in the 2016 vintage [35]. Among the different varieties harvested in the 2016 vintage (Mazuelo, Tempranillo, Garnacha, and Graciano), the stems of Mazuelo grapes were chosen because they presented the highest antioxidant capacity and phenolic content. The Mazuelo grapes were harvested in the optimum stage of ripeness for winemaking. The grape stems were stored at −20 °C until the moment of extraction (July and August 2018). For extraction, the grapes stems were oven-dried at 25 °C (stem dry matter, 22.9%), ground, and sieved (ϕ < 0.3 mm). The extract was obtained after macerating the ground and sieved stem in 50% *v*/*v* ethanol/water, with a 1:100 (*w*/*v*) ratio and at 40 °C for 24 h. Then it was centrifuged, filtered, and lyophilised; the extraction yield was 44.4%. The yield was calculated by dividing the amount of extract obtained by the amount of total grape stem used for the extraction and multiplying by 100. Vinetan^®^ is a commercial extract from vine wood, kindly provided by Actichem S.A. (Mountauban, France). This extract is a brown powder obtained with a mixture of ethanol and water and has a high concentration of stilbenes.

### 2.2. Wine Samples

The entire winemaking process was performed in the winery of the Navarra Viticulture and Oenological Research Station (EVENA). Grapes of the Tempranillo variety from the 2018 vintage were destemmed and crushed. Next, this was taken to 25 L stainless steel tanks, where alcoholic and malolactic fermentations were carried out at a controlled temperature. Six stainless steel tanks were used, two for the control wines, two for the samples with grape stem extract addition, and two for the wines with commercial extract (Vinetan^®^) addition. To the control musts, 60 mg/L of SO_2_ were added, while the SO_2_ dose in the samples, with the addition of extracts, was 20 mg/L. The amount of extract added, for both the grape stem and the Vinetan^®^, was 100 mg/L. At this time, commercial yeasts were inoculated (*Saccharomyces cerevisae* var. Bayanus, Oenoferm^®^ Be-Red, Erbslöh) in the musts to start the alcoholic fermentation. Maceration was carried out during alcoholic fermentation, with periodic pumping over. The alcoholic fermentation lasted 12 days and then the wine was racked to another tank and the grape pomace was pressed. In each case, the press wine was mixed with the corresponding free run wine. Next, commercial lactic acid bacteria were inoculated in order to initiate the malolactic fermentation, which also lasted 12 days. Once the malolactic fermentation had finished, the wines were racked again and then SO_2_ was added to all of the samples: 6 g/hL to the control wines and 2 g/hL to the wines vinified with extracts. A month later, another racking was carried out to eliminate the settled lees and, in this case, SO_2_ only was added to the control wines (3 g/hL). From this moment on, the wines were kept at 4 °C for a month to facilitate tartaric stabilization. After that month, a new addition of SO_2_ was made to the control wines (4 g/hL) and then all the wines obtained were bottled. Three different samples from each of the stainless steel tanks were taken before the alcoholic fermentation (must), halfway through the alcoholic fermentation (50% of sugars consumed), at the end of the alcoholic fermentation, at the end of the malolactic fermentation, before bottling, and after one year of bottle aging in cellar conditions. Upon obtaining, samples were stored at −20 °C until analysis, and all of them (six samples per treatment and time point) were analysed at the same time.

### 2.3. Determination of Antioxidant Capacity of the Wines

The antioxidant capacity of plant-derived extracts is due to a combination of the activities of several antioxidant compounds, and therefore, it is necessary to properly assay it using methods with different mechanisms of action. In addition, phenolic compounds usually present different antioxidant features simultaneously. For that reason, the antioxidant capacity was determined by three different methods: ABTS radical scavenging assay, DPPH radical scavenging assay, and FRAP (Ferric Ion Reducing Antioxidant Power). The ABTS (2,2′-azinobis(3-ethylbenzothiazoline-6-sulphonic acid)) method was based on the method described by Re et al. [36]. Briefly, a solution of ABTS 7 mM with potassium persulfate 2.45 mM was prepared, and the mixture was left in darkness for 16 h (ABTS^●+^ radical cation). A calibration curve was made from a 5 mM solution of Trolox, ranging from 0.05 to 2.11 mM. For sample measurement, 30 µL of the Trolox standard solution, the wine sample (previously diluted 1:7 or 1:15 with methanol), or the extract (previously dissolved in methanol at a concentration of 1 mg/mL) were mixed with 2.97 mL of ABTS^●+^ solution. After 30 min in darkness, absorbance was measured at 734 nm with a UV/Vis spectrometer (Jenway 7315, Staffordshire, UK).

The DPPH (2,2-diphenyl-1-pycrilhydracyl) assay was based on the method outlined by Brand-Williams et al. [37]. A standard solution of 24 mg of DPPH in 100 mL methanol was prepared and then, it was diluted in methanol until obtaining an absorbance of 0.9 ± 0.1 at 517 nm. For the calibration curve, Trolox was used in different concentrations ranging from 0.05 mM to 0.62 mM. For sample measurement, 150 µL of the Trolox standard solution, the wine sample (previously diluted 1:7 or 1:15 with methanol), or the extract (previously dissolved in methanol at a concentration of 1 mg/mL) were mixed with 2.85 mL of the DPPH solution. After 30 min in darkness, the antioxidant capacity was determined by measuring the absorbance at 517 nm.

Finally, the antioxidant capacity of the samples was also determined by the FRAP assay proposed by Benzie and Strain [38]. Known concentrations of Trolox, in the range of 0.05–1.18 mM, were used for preparing the calibration curve. For sample measurement, 150 µL of the Trolox standard solution, the wine sample (previously diluted 1:15 or 1:25 with methanol), or the extract (previously dissolved in methanol at a concentration of 0.5 mg/mL) were mixed with 2.85 mL of FRAP reagent (acetate buffer 300 mM:2,4,6-tris(2-pyridyl)-s-triazine 9.99 mM: FeCl_3_·6H_2_O 20 mM, 10:1:1). Mixtures were left for 30 min and then absorbance was measured at 595 nm.

The results of antioxidant capacity were expressed as mM of Trolox in must and wine samples, and as mmol Trolox/g in extract samples. All the antioxidant capacity determinations were made in triplicate.

### 2.4. Spectrophotometric Determination of Total Anthocyanins, Phenolic Content, and Flavonoids

The concentration of total monomeric anthocyanins was determined using the differential pH method proposed by the AOAC and described by Lee et al. [39]. The must and wine samples were diluted 1:7 and 1:10 respectively with the corresponding buffer solution (pH 1—0.025 M potassium chloride buffer and pH 4.5—0.4 M sodium acetate buffer), and the absorbance was measured at two wavelengths (520 nm and 700 nm) for each pH. The amount of total anthocyanin pigment was expressed as malvidin-3-glucoside (mg/L). In all cases, the analyses were performed in triplicate.

The total phenolic content was analysed using the Folin–Ciocalteu method as described by Singleton et al. [40]. The standard used for the calibration curve was gallic acid, ranging between 0.2 and 5.08 mM. For sample measurement, 0.1 mL of the gallic acid standard, the wine (previously diluted 2–4 times with methanol), or the extract (previously dissolved in methanol at a concentration of 5 mg/mL) were mixed with 0.5 L of Folin–Ciocalteu reagent, 7.9 mL of deionised water, and 1.5 mL of Na_2_CO_3_ (20% *w*/*w*), and the resulting solutions were left for 2 h in darkness. The absorbance was measured at 765 nm in a UV-Vis spectrophotometer (Jenway, Staffordshire, UK). The results of total phenolic content were expressed as mM of gallic acid in must and wine samples, and as mmol gallic acid/g in extract samples. In all cases, the samples were analysed in triplicate.

The total flavonoid content was determined by the colorimetric method of aluminium chloride using a solution of 2% AlCl_3_ in 5% acetic acid [41]. The calibration curve was performed using a quercetin commercial standard in concentrations between 3–25 ppm. Must samples were diluted 1:5 or 1:7 with methanol. After that, 1.5 mL of quercetin standard solution or sample were mixed with 1.5 mL of the AlCl_3_ solution, and the resulting solutions were left for 30 min in darkness. Then, absorbance was measured on a Jenway UV-Vis spectrophotometer at 420 nm. The results were expressed as mg of quercetin/L. In all cases, the samples were analysed in triplicate.

### 2.5. Identification and Quantification of Phenolic Composition by HPLC-DAD

Identification and quantification of the phenolic compounds present in the extracts and in the wine samples were carried out using high-performance liquid chromatography equipped with two 510 pumps, a 717 Plus autosampler, and a 996 photodiode array detector (Waters Div., Milford, MA, USA). A Zorbax Eclipse Plus C18 reversed-phase column (250 × 4.6 mm, particle size 5 µm, Agilent, Santa Clara, CA, USA) was used. For the analyses of the extracts (both commercial and obtained in our laboratory), around 50 mg of each extract were weighed and dissolved in 10 mL of methanol with the aid of an ultrasonic bath (JP Selecta, Barcelona, Spain). An additional 10-time dilution step was necessary for commercial extracts. The must and wine samples were centrifuged before chromatographic analyses to remove solid particles in suspension, and 3 mL of each sample was lyophilised in a Cryodos-50 lyophiliser (Telstar, Spain). The resulting powder was reconstituted in 0.6 mL of methanol:water (50:50 *v*/*v*) with 1% HCl. Finally, all the samples were filtered through 0.45 μm PTFE syringe filters prior to their analysis. For the chromatographic analyses, a modified method of Barros et al. [42] was used. Two mobile phases, A (water: 85% formic acid, 99:1 *v*/*v*) and B (acetonitrile: 85% formic acid, 99:1 *v*/*v*) were used. The flow rate was 1 mL/min using the following linear gradient scheme (t in min; % A): (0; 95%), (15; 85%), (22; 80%), (25, 80%), (35, 70%), (45; 50%), (50, 5%), (55, 95%), (60, 95%). All the HPLC quality solvents were from Scharlab (Barcelona, Spain). The injection volume was 40 μL and the column temperature was 30 °C. The identification of the different compounds was performed by the double coincidence of the UV-Vis spectrum at the characteristic wavelength of each compound, and the retention time of its corresponding standard. Quantification was carried out using calibration curves for each compound analysed. In the case of anthocyanins, we used several standards such as malvidin-3-glucoside, cyanidin, and keracyanin, but only malvidin-3-glucoside was positively identified. For the unidentified anthocyanins (A–E) the malvidin-3-glucoside calibration curve was used. All the standards used were from Sigma-Aldrich (Madrid, Spain), with the exception of malvidin-3-glucoside (Oenyn chloride, Extrasynthese, Genay, France).

### 2.6. Oenological Parameters of Wines

General parameters were measured before bottling following the OIV compendium of international methods of wine and must analysis [43] in a laboratory accredited by ENAC (UNE-EN ISO/IEC 17025:2017).

### 2.7. Sensory Analysis

The wines obtained before bottling and one year after bottling were subjected to a blind tasting. The procedure was conducted following the OIV tasting sheet for still wines [44]. Briefly, the different phases of the sensory analysis were evaluated on a total score of 100 points by at least seven trained panellists. Those phases were visual, nose, taste, and harmony (overall judgement). The specific parameters evaluated in each phase were the following: visual—Colour and limpidity and brightness; nose—Genuineness (absence of defects), positive intensity (or quantity of aroma), quality (complexity, richness of the aromatic palette); taste—Genuineness, positive intensity, harmonious persistence (length of residual olfacto-gustatory sensation), quality (based on richness of the taste, complexity, and structure); harmony—Overall appraisal of wine.

The assessment took place in a standard sensory analysis laboratory (ISO 8589:2010), equipped with separate booths with controlled temperature (20–22 °C) and light. Normalised glasses (UNE 87022:1992) containing 50 mL of wine were used.

### 2.8. Statistical Analysis

Data are expressed as the mean ± standard deviation (S.D.). Data from antioxidant measurements were subjected to a hierarchical linear model in order to incorporate repeated measurements in the study. On the other hand, to investigate statistical differences in results obtained after the sensory analyses of the different wines, we consider an ANOVA with blocking, where tasters are the blocks, and residuals satisfy normality conditions in all the cases. In all cases, *p* ≤ 0.05 was considered to be statistically significant. All data processing was conducted by using SPSS^®^ statistical software (IBM^®^ SPSS^®^ Statistics for Windows Version 25.0. IBM Corp, Armonk, NY, USA)

## 3. Results and Discussion

### 3.1. Extracts

Table 1 shows the composition of the commercial extract Vinetan^®^ and the Mazuelo grape stem extract. As can be seen, in the former, resveratrol and viniferin were found in very important quantities. However, in grape stems, the concentration of these stilbenes was much lower than those of the commercial extract, but also included other phenolic compounds with high antioxidant potential such as catechin, quercetin, a quercetin derivative, gallic acid, malvidin-3-glucoside, and an unidentified anthocyanin. Regarding the antioxidant capacity of both extracts, the results obtained by the DPPH and FRAP methods were similar, but Vinetan^®^ showed higher antioxidant capacity when samples were quantified by the ABTS method. DPPH and ABTS are considered electron transfer-based assays, but hydrogen atom transfer also takes place in both methods, although in the DPPH assay, this mechanism is more marginal [45]. In the FRAP assay, there are no free radicals implicated in the reaction, but rather it is based on the ability of the antioxidants to reduce ferric iron (Fe^3+^) to ferrous iron (Fe^2+^). Although ABTS and DPPH share the same mechanism of action, the radical site in the DPPH molecule is located inside a reaction cage formed by the two phenyl rings orthogonal to each other, and the pycril ring angled at about 30° with two nitro groups oriented above and below the radical site [46]. Therefore, steric accessibility is a limiting factor in the DPPH reaction, which could explain the lower values observed when applying this method with respect to the ABTS assay.

It is noteworthy that the concentration of stilbenes in the Mazuelo grape stem extract is around 100 times lower than that of the commercial extract, while the antioxidant capacity of both extracts is of the same order. This difference suggests that the antioxidant potential of flavonoids is much greater than that of the stilbenes. In previous work, it was also found that the antioxidant capacity of resveratrol and viniferin is low in comparison to that of quercetin or malvidin-3-glucoside, probably due to structural features of these molecules [35].

Table 2 shows the general oenological parameters of the different wine samples before bottling. As can be seen, wines treated with extracts presented free and total SO_2_ concentrations much lower than those of the control wine. The ratio between free SO_2_ and total SO_2_ was 0.5 for the control sample and also ~0.5 for samples in the presence of Vinetan^®^ and grape stem extract. The rest of the parameters were found within the normal values and were of the same order in the three types of samples tested. In view of this, it can be considered that the partial replacement of SO_2_ with vine wood (Vinetan^®^) and grape stem extracts did not have a significant impact on these parameters.

### 3.2. Antioxidant Capacity of Wine Samples

Table 3 shows the antioxidant capacity results of the different samples measured by the ABTS, DPPH, and FRAP methods. These results were subjected to a hierarchical linear model in order to detect significant differences among the treatments considering repeated measurements. The results of these statistical analyses are shown in Tables S1–S3 of the Supplementary Material. The highest values were found when the ABTS method was used, followed by FRAP and DPPH. Although some significant differences were observed between the three treatments throughout winemaking, no statistical differences from control samples were found after 12 months of storage in a bottle. Probably, this is because more than 50% of the SO_2_ added to the musts is combined with sugars, carbonyls, and phenolic compounds during the winemaking process [47] and therefore, loses its antioxidant capacity [48].

These results are in agreement with the conclusions obtained by Salaha et al. [49], who also found no significant differences in the antioxidant activity between wines of different varieties treated with normal doses of SO_2_ and the same wines treated with reduced doses of SO_2_ combined with black radish extracts (*Rafhanus niger*) and ascorbic acid.

### 3.3. Phenolic Composition of Wine Samples

**Figure 1:**
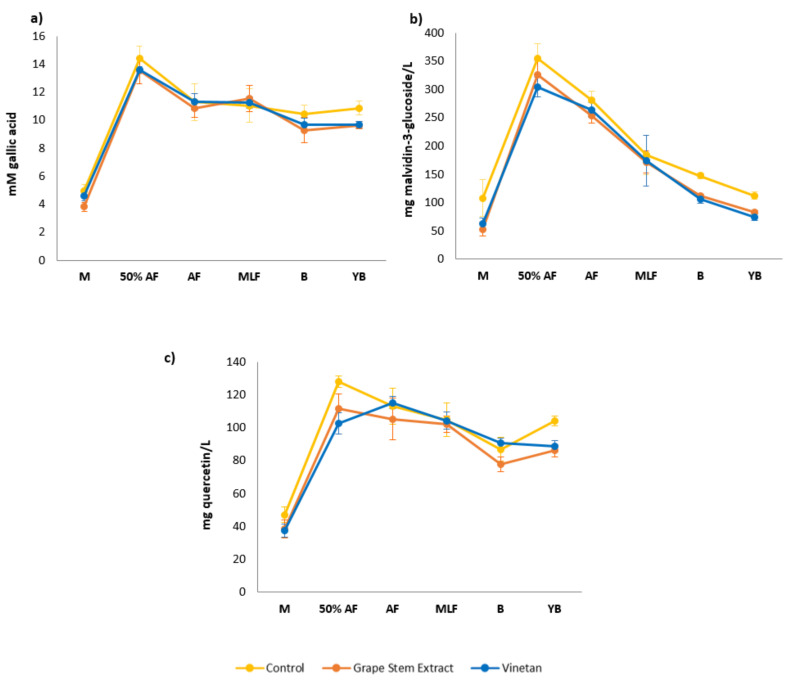
Concentration of (**a**) total phenolic content (mM gallic acid), (**b**) total anthocyanins (mg malvidin-3-glucoside/L), and (**c**) total flavonoids (mg quercetin/L), obtained by spectrophotometric methods. M = Must; 50% AF = Half Alcoholic Fermentation; AF = Final Alcoholic Fermentation; MLF = Final Malolactic Fermentation; B = bottling; and YB = one year after bottling (Mean ± SD, *n* = 6).

Phenolic compounds have an important role in the quality of wines, as they are the main compounds responsible for its colour, astringency, and antioxidant capacity [50]. These compounds can be modified by different reactions that occur during winemaking and ageing, which involves changes in the sensory quality of wines [51]. Figure 1 represents the evolution of the concentration of total polyphenols (a), anthocyanins (b), and flavonoids (c) throughout the winemaking process in function of the treatment applied to the must. These measurements have been conducted by spectrophotometric methods, so they represent the total content of each group of compounds. As can be seen, the evolution of the concentration values of the three components during the vinification process is similar in the three types of analysed samples and corresponds with the normal behaviour of these compounds [3,52,53].

It is worth mentioning the higher levels of total anthocyanins found in the control samples in the initial phase of vinification and also in wines in the bottling and ageing phase, in comparison to samples treated with extracts. This difference from the beginning of the vinification process could have been attributed to the higher concentration of SO_2_ used in the control sample, since this compound breaks the grape cells favouring the extraction of anthocyanins [3,54], reducing their rate of loss and, to a lesser extent, their polymerisation [55]. However, a difference of 40 mg/L of SO_2_ could not be significant enough to induce those changes in the extraction of anthocyanins from grape skin. For this reason, it is more likely that the cause of such differences is due to the condensation reactions between free anthocyanins and the tannins of the extracts. These reactions reduce the amount of free anthocyanins in wine, which are the anthocyanins detected by the analytical method used in the present work (polymerised anthocyanin pigments are not measured by this method [39]). The total content of polyphenols and flavonoids one year after bottling was higher for the control wines in comparison to the wines containing extracts and reduced doses of SO_2_ (Figure 1). On the other hand, in the present study, no differences were found between the values of total polyphenols, anthocyanins, and flavonoids in wines treated with grape stem extract and the wines treated with vine wood extract (Vinetan^®^). This may be explained by its similar antioxidant potential, despite its different composition.

These results are in agreement with the observations made by other authors, who found higher anthocyanin, polyphenol, or flavonoid levels in wines treated with standard doses of SO_2_ than in wines treated with other alternatives, such as black radish extracts and ascorbic acid [49], hydroxytyrosol [22], or a commercial vine wood extract called Vineatrol^®^, similar to one of the extracts used in the present work [33].

**Figure 2:**
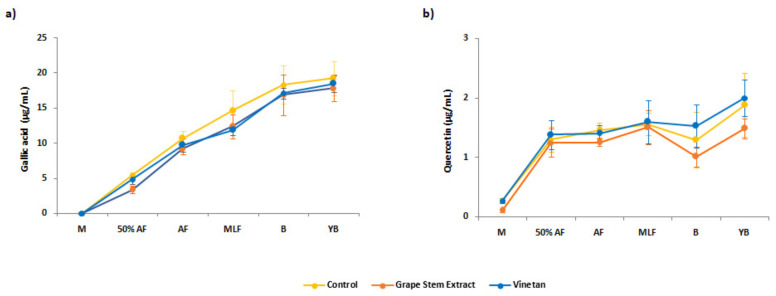
Concentration (µg/mL) of gallic acid (**a**) and quercetin (**b**) during the winemaking process of the control samples and samples with the addition of grape stem extract or commercial extract (Vinetan^®^). Results were obtained by HPLC-DAD analyses. M, must; 50%AF, 50% alcoholic fermentation; AF, end of alcoholic fermentation; MLF, end of malolactic fermentation; B, bottling; YB, one year after bottling (Mean ± SD, *n* = 6).

Once the total content of polyphenols was measured, the composition of different individual polyphenols of the wine samples was determined by HPLC-DAD analyses. Therefore, gallic acid, quercetin, malvidin-3-glucoside, and five different unidentified anthocyanins were quantified in the different wine samples of the present work. The first of them, gallic acid, is considered the most important phenolic compound, as it is the precursor of all hydrolysable tannins and it is also included within condensed tannins [56]. At the beginning of the vinification process, gallic acid was not detected in any of the musts analysed in the present study (see Figure 2a)

During vinification, gallic acid was detectable and its concentration increased progressively in all samples. This behaviour was similar to that described by other authors [57,58] in the vinification of different grape varieties. The concentration of gallic acid (Figure 2a) was similar for the three types of samples analysed during the vinification process and remained stable throughout one year of aging. In view of these results, it can be considered that the partial substitution of SO_2_ by the extracts tested in this study did not influence the extraction and evolution of this compound during winemaking and subsequent preservation of the wine. Regarding quercetin, its initial concentration was low for all the analysed musts, being slightly lower in the sample treated with grape stem extract (Figure 2b). The concentration of this compound increased in the first half of the alcoholic fermentation in all samples, without differences among them, and remained stable in the rest of the winemaking stages. After 12 months in a bottle, all wines showed a slight increase tendency in the quercetin concentration, which could be due to the hydrolysis of the glycosylated forms of the flavonoid [59].

Table 4 shows the results obtained from the quantitative analysis of the anthocyanins identified in the samples. The main anthocyanin in all cases was malvidin-3-glucoside, which is the most abundant in the majority of grape varieties. However, the concentration of the rest of anthocyanins can be variable among the different grape varieties [60].

In all cases, the anthocyanin extraction took place mainly during maceration and alcoholic fermentation, which is in agreement with the results obtained by other authors [61] and coincides with the usual behaviour of this kind of compounds. As can be seen in Table 4, the concentration of the different anthocyanins decreased during malolactic fermentation, although the losses of these compounds were greater during aging in all cases except for the unknown anthocyanin C, which remained stable. Similarly, Marquez et al. [59] also found decreases in the anthocyanin content during the aging of wines from Merlot, Syrah, and Tempranillo grape varieties. When comparing the three different treatments (control, treated with grape stem extracts, and treated with Vinetan^®^), at the beginning of the vinification process the highest anthocyanin concentrations were found in control samples. This could also be attributed to the condensation reactions between free anthocyanins and the tannins of the extract, since these reduce the amount of free detectable anthocyanins in wines treated with extracts. At the end of the study, only the main anthocyanins (malvidin-3-glucoside and anthocyanins A and B) showed concentration levels slightly higher in control wines. No differences were found between the wines treated with any of the extracts.

### 3.4. Sensory Analysis

A sensorial analysis of the samples subjected to the different treatments was conducted according to the criteria of the OIV tasting sheet [44]. The analysis was conducted at the end of the vinification process (before bottling) and one year after bottling. Table 5 and Table 6 show the results obtained.

As can be seen, all wines presented good tasting scores, exceeding 77 points out of 100 according to the OIV standards [44]. Wines treated with both Vinetan^®^ and grape stem extract presented before bottling significantly higher scores for the visual characteristics in comparison to control wines. This difference could also be attributed to the condensation reactions between free anthocyanins and the tannins of the extracts, as it is well known that such reactions involve colour intensification [3]. The rest of the parameters, including overall judgement, did not present statistically significant differences among the three wines. After one-year of evolution in a bottle wines treated with both Vinetan^®^ and grape stem extract before bottling had higher scores for the olfactory characteristics in comparison to control wines. The rest of the parameters, including overall judgement, did not present statistically significant differences among the three wines.

All these results suggest that the partial replacement of SO_2_ by grape stem or vine wood extracts could lead to wines with equal or even better sensory quality results, both at the end of the vinification process and during aging in a bottle.

It is worth mentioning that, in both sensory analyses, tasters highlighted in their comments a greater body, potency in mouth, and astringency of wines treated with extracts in comparison with the control one, although the data did not reflect that preference. Moreover, none of the tasters found any defect in any of the samples tested.

Presently, the addition of phenolic compounds as substitutes of SO_2_ in winemaking is not carried out in wineries. This is mainly due to the negative impact that they can have on the sensory characteristics of the resulting wines [15]. In addition, although the effect can be more likely observed in white wines, the absence of SO_2_ in wines can also increase the typical oxidation aromas [25], even if polyphenolic SO_2_ substitutes such as hydroxytyrosol are used [22]. However, the results obtained in the present work revealed that the grape stem and vine wood extracts could be used as SO_2_ substitutes in winemaking without negatively affecting the sensorial quality of the resulting red wines. This is the first time that it is demonstrated that grape stem extract is a good alternative for this purpose. However, additional tests should be carried out in order to confirm this hypothesis and evaluate the impact of the use of different extract doses to totally or partially replace SO_2_ on the final quality of wines.

## 4. Conclusions

In the present study, a commercial vine wood extract, called Vinetan^®^, and a grape stem extract obtained in our laboratory, were tested as alternatives to reduce the amount of SO_2_ used in the fermentation stage of the winemaking of Tempranillo wines. Control musts showed greater antioxidant activity and free anthocyanin content than the musts treated with any of the extracts under study. After 12 months of storage in a bottle, no differences in antioxidant capacity were found among the samples, but the total anthocyanin, polyphenol, and flavonoid contents were slightly higher in control wines than in those treated with extracts. In general, no large quantitative differences were observed in the phenolic composition of the samples subjected to different treatments. Sensory analyses before bottling and one year after bottling, also confirmed that the use of extracts as partial substitutes for SO_2_ could lead to wines with organoleptic properties similar or even better than control ones. Therefore, the addition of extracts obtained from the by-products of the wine industry, such as grape stems, may be an appropriate strategy to reduce or even eliminate the SO_2_ content in red wines. Further studies should be performed in order to optimise the dosage of the extract necessary to eliminate the use of SO_2_ in winemaking.

## Figures and Tables

**Table 1:** Phenolic composition (mg/g extract) and antioxidant capacity of Vinetan^®^ and the Mazuelo stem extract used in this study (Mean ± SD).

Composition and Antioxidant Capacity	Vinetan^®^	Grape Stem Extract
Resveratrol	24.6 ± 2.9	0.27 ± 0.02
Viniferin	61.0 ± 5.5	0.54 ± 0.03
Gallic acid	nd	0.19 ± 0.03
(+)-Catechin	nd	0.87 ± 0.09
Quercetin	nd	0.07 ± 0.00
Quercetin-derivative ^1^	nd	0.87 ± 0.06
Malvidin-3-glucoside	nd	0.13 ± 0.01
Unknown anthocyanin ^2^	nd	0.14 ± 0.01
Total phenolic content ^3^	275.6 ± 27.2	85.1 ± 1.7
Antioxidant activity ^4^		
ABTS	1.93 ± 0.31	0.71 ± 0.01
DPPH	0.48 ± 0.07	0.45 ± 0.05
FRAP	0.49 ± 0.05	0.40 ± 0.03

^1^ expressed as quercetin-3-glucoside; ^2^ expressed as malvidin-3-glucoside; ^3^ expressed as mg gallic acid/g extract; ^4^ expressed as mmol Trolox/g extract; nd = not detected.

**Table 2:** Oenological parameters of all the wines under study before bottling (Mean ± SD).

Oenological Parameters	Control	Vinetan^®^	Grape Stem Extract
pH	4.07 ± 0.02	3.99 ± 0.02	4.01 ± 0.09
Total acidity (g/L) ^1^	3.3 ± 0.1	3.7 ± 0.0	3.5 ± 0.1
Volatile acidity (g/L) ^2^	0.5 ± 0.1	0.6 ± 0.0	0.6 ± 0.0
Alcohol content (%, *v*/*v*)	15.4 ± 0.3	15.4 ± 0.2	15.4 ± 0.2
Sugar content (g/L)	<2.5	<2.5	<2.5
Free SO_2_ (mg/L)	28 ± 8	<7	<7
Total SO_2_ (mg/L)	56 ± 18	<15	<15
Calcium (mg/L)	37 ± 0	34 ± 1	35 ± 2
Magnesium (mg/L)	86 ± 4	86 ± 4	84 ± 4
Potassium (mg/L)	1211 ± 42	1073 ± 28	980 ± 104

^1^ expressed as tartaric acid; ^2^ expressed as acetic acid.

**Table 3:** Antioxidant capacity measured by ABTS, DPPH and FRAP assays of must (M), wine at 50% alcoholic fermentation (50% AF), end of alcoholic fermentation (AF), end of malolactic fermentation (MLF), bottling (B), and one year after bottling (YB) in control samples, samples with Vinetan^®^ and wines with grape stem extract (Mean ± SD, *n* = 6).

Assay	Sample	Control	Vinetan^®^	Grape Stem Extract
ABTS (mM Trolox)	M	7.1 ± 0.8 a	5.2 ± 0.4 b	4.8 ± 0.7 c
	50% AF	21.0 ± 1.7 a	23.1 ± 1.4 b	19.5 ± 0.9 a
	AF	22.9 ± 2.4 a	22.5 ± 2.0 a	20.3 ± 2.2 a
	MLF	19.8 ± 2.7 a	21.2 ± 2.0 a	18.8 ± 3.4 a
	B	13.9 ± 0.9 a	12.3 ± 0.5 b	12.3 ± 0.5 b
	YB	13.6 ± 1.0 a	13.1 ± 0.3 a	12.1 ± 1.5 a
DPPH (mM Trolox)	M	2.8 ± 0.2 a	2.3 ± 0.2 b	2.0 ± 0.2 c
	50% AF	6.9 ± 0.4 a	6.3 ± 0.6 b	7.3 ± 0.4 a
	AF	6.5 ± 0.2 a	6.3 ± 0.1 a	6.1 ± 0.5 b
	MLF	6.8 ± 0.6 a	6.5 ± 0.2 a	6.6 ± 0.2 a
	B	6.5 ± 0.3 a	5.9 ± 0.3 b	6.1 ± 0.2 b
	YB	6.6 ± 0.5 a	6.5 ± 0.2 a	6.3 ± 0.4 a
FRAP (mM Trolox)	M	4.7 ± 0.4 a	3.0 ± 0.3 b	2.8 ± 0.6 c
	50% AF	13.1 ± 0.6 a	14.1 ± 1.0 b	12.0 ± 0.8 c
	AF	13.1 ± 0.7 a	11.5 ± 0.6 b	11.5 ± 1.0 b
	MLF	11.0 ± 1.9 a	12.3 ± 2.0 a	12.0 ± 0.8 a
	B	8.9 ± 0.7 a	6.7 ± 1.0 b	7.8 ± 0.5 c
	YB	7.7 ± 1.0 a	8.7 ± 0.3 a	7.3 ± 1.1 a

Different letters in the same row indicate significant differences (*p*-values can be found in Tables S1–S3 of the Supplementary Material).

**Table 4:** Anthocyanin concentration (µg/mL) in control samples and samples with addition of grape stem extract or commercial extract (Vinetan^®^) during winemaking (M = must; 50% AF = 50% alcoholic fermentation; AF = end of alcoholic fermentation; MLF = end of malolactic fermentation; B = bottling; YB = one year after bottling) (Mean ± SD, *n* = 6). Results were obtained by HPLC-DAD analyses. Letters, A-E are some unidentified anthocyanins, other than malvidin-3-glucoside.

Treatment		Malvidin-3-Glucoside	A	B	C	D	E
**Control**	M	20.1 ± 1.2	9.3 ± 1.3	7.0 ± 0.6	0.2 ± 0.0	0.6 ± 0.0	2.0 ± 0.1
	50%AF	101.9 ± 7.9	19.3 ± 3.2	20.6 ± 3.3	1.5 ± 0.2	3.6 ± 0.6	14.3 ± 1.9
	AF	111.4 ± 4.8	13.0 ± 0.6	17.9 ± 0.8	1.3 ± 0.1	1.7 ± 0.2	11.7 ± 0.8
	MLF	60.6 ± 34.4	6.3 ± 3.8	9.6 ± 5.8	0.8 ± 0.4	0.9 ± 0.4	5.9 ± 2.9
	B	64.5 ± 9.7	6.4 ± 0.6	10.9 ± 1.6	1.0 ± 0.4	0.5 ± 0.3	5.2 ± 0.9
	YB	44.6 ± 6.0	4.8 ± 0.5	7.4 ± 0.9	0.9 ± 0.3	0.4 ± 0.1	3.4 ± 0.5
**Grape Stem Extract**	M	11.2 ± 0.9	1.5 ± 0.2	1.7 ± 0.1	<0.1	0.2 ± 0.0	1.0 ± 0.2
	50%AF	91.7 ± 12.6	14.7 ± 2.0	16.1 ± 2.2	1.4 ± 0.2	3.2 ± 0.4	12.1 ± 2.0
	AF	113.2 ± 16.0	10.8 ± 0.7	15.7 ± 1.2	1.2 ± 0.3	2.1 ± 0.2	10.1 ± 0.9
	MLF	78.3 ± 19.7	7.1 ± 1.4	11.2 ± 1.8	1.0 ± 0.1	1.2 ± 0.1	7.6 ± 1.8
	B	57.6 ± 2.9	5.2 ± 0.6	8.7 ± 0.6	0.9 ± 0.1	0.4 ± 0.1	4.8 ± 0.4
	YB	34.8 ± 2.3	3.6 ± 0.4	5.4 ± 0.5	0.8 ± 0.1	0.2 ± 0.1	2.9 ± 0.3
**Vinetan^®^**	M	12.1 ± 1.3	1.9 ± 0.7	2.1 ± 0.6	<0.1	0.2 ± 0.0	1.1 ± 0.2
	50%AF	88.7 ± 9.8	15.3 ± 1.7	16.4 ± 1.7	1.6 ± 0.2	2.3 ± 0.3	12.5 ± 1.9
	AF	102.0 ± 10.5	11.6 ± 1.3	16.5 ± 1.8	1.2 ± 0.1	2.2 ± 0.3	9.9 ± 1.3
	MLF	63.6 ± 18.8	6.3 ± 2.4	9.7 ± 3.4	0.8 ± 0.2	1.3 ± 0.3	5.8 ± 2.0
	B	53.9 ± 8.1	5.2 ± 1.4	8.5 ± 1.9	0.9 ± 0.2	0.4 ± 0.2	4.1 ± 0.9
	YB	31.2 ± 3.6	3.5 ± 0.7	5.2 ± 0.9	0.7 ± 0.1	0.2 ± 0.1	2.4 ± 0.3

**Table 5:** Results of the sensory test of wines produced with only SO_2_ at usual concentrations (control), or with grape stem or Vinetan^®^ extracts and reduced doses of SO_2_, before bottling (Mean ± SD, *n* = 18).

Sensory Tests	Control	Vinetan^®^	Grape Stem Extract
**Visual**	10.7 ± 1.3 a	12.1 ± 0.2 b	11.7 ± 0.9 b
**Nose**	24.4 ± 2.4 a	24.6 ± 1.6 a	24.1 ± 1.5 a
**Taste**	34.6 ± 4.0 a	35.5 ± 2.5 a	34.9 ± 2.6 a
**Harmony** **(Overall Judgement)**	9.3 ± 0.7 a	9.4 ± 0.5 a	9.4 ± 0.5 a
**Total Score**	79.0 ± 7.2 a	81.6 ± 3.7 a	80.1 ± 4.1 a

Different letters in the same row indicate significant differences (Tukey test: *p*-values < 0.05).

**Table 6:** Results of the sensory test of wines produced with only SO_2_ at usual concentrations (control), or with grape stem or Vinetan^®^ extracts and reduced doses of SO_2_, preserved for 12 months in a bottle (Mean ± SD, *n* = 14).

Sensory Tests	Control	Vinetan ^®^	Grape Stem Extract
**Visual**	10.9 ± 2.2 a	11.9 ± 1.5 a	11.4 ± 1.2 a
**Nose**	22.6 ± 2.8 a	25.4 ± 2.1b	24.1 ± 2.3 ab
**Taste**	34.4 ± 3.9 a	34.9 ± 3.4 a	34.9 ± 3.1 a
**Harmony** **(Overall Judgement)**	9.3 ± 0.6 a	9.4 ± 0.5 a	9.4 ± 0.6 a
**Total Score**	77.1 ± 8.3 a	81.6 ± 5.5 a	79.9 ± 5.8 a

Different letters in the same row indicate significant differences (Tukey test: *p*-values < 0.05).

## Supplementary Materials

The following are available online at https://www.mdpi.com/2218-273X/10/10/1369/s1, Table S1: Estimations of the hierarchical linear model for the ABTS results, Table S1: title, Table S2: Estimations of the hierarchical linear model for the DPPH results, Table S3: Estimations of the hierarchical linear model for the FRAP results, Table S4: Estimations of the linear model for the results of the sensory analysis of wines before bottling, Table S5: Estimations of the linear model for the results of the sensory analysis of wines after for 12 months in bottle.
