# Supplementary material for: Reducing SO2 Doses in Red Wines by Using Grape Stem Extracts as Antioxidants
Source: Biomolecules. 2020 Sep 25;10(10):1369. doi: 10.3390/biom10101369 (PMC7599755; doi:10.3390/biom10101369)
Supplement: Supplementary file 1 [file biomolecules-10-01369-s001.pdf]

Supplementary Material

A. Statistical analyses of antioxidant capacity data

Table 1. Estimations of the hierarchical linear model for the ABTS results.

	Control[a]	Vinetan	Grape Stem Extract
**M**	7.15***	-1.91***	-2.37***
**50%AF**	20.98***	2.14**	-1.48
**AF**	22.86***	-0.32	-2.58
**MLF**	19.84***	1.35	-1.04
**B**	13.85***	-1.54***	-1.52**
**YB**	13.56***	-0.45	-1.46

[a] Intercept.

p-value: *** <0.001; **<0.025; *<0.05.

Table 2. Estimations of the hierarchical linear model for the DPPH results.

	Control[a]	Vinetan	Grape Stem Extract
**M**	2.83***	-0.52***	-0.87***
**50%AF**	6.95***	-0.63*	0.31
**AF**	6.53***	-0.2	-0.46***
**MLF**	6.77***	-0.25	-0.14
**B**	6.48***	-0.54***	-0.34**
**YB**	6.64***	-0.18	-0.37

[a] Intercept.

p-value: *** <0.001; **<0.025; *<0.05.

Table 3. Estimations of the hierarchical linear model for the FRAP results.

	Control[a]	Vinetan	Grape Stem Extract
**M**	4.67***	-1.72***	-1.85***
**50%AF**	13.10***	1.01*	-1.06**
**AF**	13.12***	-1.59***	-1.59***
**MLF**	10.97***	1.35	1.08
**B**	8.92***	-2.21***	-1.14**
**YB**	7.71***	0.97	-0.42

[a] Intercept.

p-value: *** <0.001; **<0.025; *<0.05.

B. Statistical analyses of sensory analyses data

Table 4. Estimations of the linear model for the results of the sensory analysis of wines before bottling.

	Control	Vinetan	Grape Stem Extract
**Visual**	10.2***	1***	1.39***
**Olfactory**	24.57***	-0.33	-0.11
**Mouthfeel**	36.1***	0.27	0.89
**General**	9.57***	0.11	0.17
**Total**	80.46***	0.43	0.06

[a] Intercept.

p-value: *** <0.001; **<0.01; *<0.05.

Table 5. Estimations of the linear model for the results of the sensory analysis of wines after for 12 months in bottle.

	Control	Vinetan	Grape Stem Extract
**Visual**	11.29***	0.57	1.07*
**Olfactory**	23.07***	1.5*	2.79***
**Mouthfeel**	34.5***	0.5	0.5
**General**	9.1***	0.14	0.07
**Total**	77.95***	2.71	4.43*

[a] Intercept.

p-value: *** <0.001; **<0.01; *<0.05.

It can be observed that linear model considers significant some coefficient whereas Tuckey analysis (Tables 5 and 6) does not, this is because Tukey analysis is a global comparative test for the means of the three treatments.
